# Supplementary material for: The impact of the COVID-19 pandemic on wellbeing and cognitive functioning of older adults
Source: Sci Rep. 2021 Feb 25;11:4636. doi: 10.1038/s41598-021-84127-7 (PMC7907111; doi:10.1038/s41598-021-84127-7)

# The impact of the COVID-19 pandemic on wellbeing and cognitive functioning of older adults

# Supplementary Information

Sarah De Pue^*^, Céline Gillebert, Eva Dierckx, Marie-Anne Vanderhasselt, Rudi De Raedt & Eva Van den Bussche

*Corresponding author

E-mail: [sarah.depue@kuleuven.be](mailto:sarah.depue@kuleuven.be)

**Supplementary Table 1**

*Overview of participants’ demographic and general characteristics.*

|  |  |  | N |
| --- | --- | --- | --- |
| Age Category | 65-69 |  | 248 |
|  | 70-74 |  | 187 |
|  | 75-79 |  | 87 |
|  | 80-84 |  | 58 |
|  | 85-89 |  | 38 |
|  | ≥90 |  | 22 |
| Gender | Male |  | 262 |
|  | Female |  | 377 |
|  | Other |  | 1 |
| Nationality | Belgian |  | 632 |
|  | Dutch |  | 5 |
|  | German |  | 1 |
|  | Moroccan |  | 2 |
| Living situation | Own or partner’s home |  | 518 |
|  | Care facility | Nursing home | 53 |
|  |  | Assisted living facility | 67 |
|  | With family |  | 1 |
|  | Other |  | 1 |
| Number of cohabitants | 0 |  | 179 |
|  | 1 |  | 345 |
|  | 2 or more |  | 116 |
| Number of contacts during the past week (excluding cohabitants) | In real life outside | 0  1  2  3 or 4  5 to 8  +9 | 108  55  81  185  142  62 |
|  | In real life inside | 0  1  2  3 or 4  5 to 8  +9 | 259  106  106  110  33  20 |
|  | By telephone | 0  1  2  3 or 4  5 to 8  +9 | 39  42  71  203  167  113 |
|  | By internet | 0  1  2  3 or 4  5 to 8  +9 | 104  43  56  145  128  157 |
| Highest educational level | No secondary school diploma |  | 81 |
|  | Secondary school diploma |  | 181 |
|  | University or high school degree |  | 331 |
|  | Other |  | 47 |
| Work situation | Retired |  | 610 |
|  | Employed |  | 14 |
|  | Other (e.g., disabled or retired with part-time job) |  | 16 |
| Monthly individual net income (in €) | 0-500 |  | 31 |
|  | 501-1000 |  | 22 |
|  | 1001-1500 |  | 161 |
|  | 1501-2000 |  | 189 |
|  | 2001-2500 |  | 109 |
|  | 2501-3000 |  | 36 |
|  | 3001-3500 |  | 9 |
|  | 3501-4000 |  | 6 |
|  | Above 4000 |  | 8 |
|  | I can’t or do not want to answer this question |  | 69 |
| Medical history | Parkinson’s disease |  | 7 |
|  | Dementia |  | 7 |
|  | Stroke |  | 9 |
|  | Diabetes |  | 53 |
|  | Epilepsy |  | 3 |
|  | None of the above |  | 563 |
| COVID-19 contagion of participant | No |  | 613 |
|  | Yes, confirmed with test |  | 7 |
|  | Yes, but not confirmed with test |  | 20 |
| COVID-19 contagion of at least one close relative or friend | No |  | 542 |
|  | Yes, confirmed with test |  | 43 |
|  | Yes, but not confirmed with test |  | 55 |
| Week when survey was completed | 19/5/2020 until 25/5/2020 |  | 285 |
|  | 26/5/2020 until 1/6/2020 |  | 106 |
|  | 2/6/2020 until 8/6/2020 |  | 140 |
|  | 9/6/2020 until 15/6/2020 |  | 70 |
|  | 16/6/2020 until 22/6/2020 |  | 39 |

**Supplementary Table 2**

*Pearson correlations between the continuous predictors (age, income, CFQ, GDS-15, LSNS-6 and BRS total scores) and the difference scores between the reports during and before the COVID-19 period for the PWI-A items and total score, activity level and sleep quality. Means (SD) for the difference scores for each level of the categorical predictors (i.e., gender, whether the participant lived alone or not and whether the participant lived in a care facility or not).*

| Difference scores with regards to | | | | | | | | | | | |
| --- | --- | --- | --- | --- | --- | --- | --- | --- | --- | --- | --- |
|  | PWI-A1 | PWI-A2 | PWI-A3 | PWI-A4 | PWI-A5 | PWI-A6 | PWI-A7 | PWI-A8 | PWI-A_total | Activity | Sleep |
| Age | .041 | -.11** | -.005 | .044 | .038 | .14*** | .068 | .17*** | .080* | -.005 | .005 |
| Income | .000 | -.019 | -.010 | -.038 | -.011 | .063 | .012 | .056 | .018 | .054 | .040 |
| CFQ | -.058 | -.011 | -.069 | -.12** | -.091* | -.15*** | -.075 | -.14** | -.13** | -.053 | -.083* |
| GDS-15 | -.47*** | -.31*** | -.30*** | -.37*** | -.32*** | -.35*** | -.38*** | -.38*** | -.50*** | -.38*** | -.31*** |
| LSNS-6 | .13** | .20*** | .14*** | .12** | .096* | .077 | .088* | .076 | .15*** | .084* | .098* |
| BRS | .11** | .089* | .14*** | .13** | .055 | .18*** | .11** | .16*** | .17*** | .13** | .18*** |
| Male | -7,21 (12.01) | -3,86 (12.07) | -1,53 (8.16) | -3,21 (10.15) | -4,58 (11.33) | -7,60 (13.89) | -8,17 (15.48) | -7,82 (12.33) | -5,50 (8.43) | -0,63 (1.58) | -0,29 (1.09) |
| Female | -11,32 (17.10) | -4,08 (10.95) | -3,79 (10.06) | -4,35 (11.70) | -8,86 (17.03) | -12,31 (17.24) | -12,81 (18.89) | -12,15 (15.91) | -8,71 (10.82) | -1,15 (1.61) | -0,26 (1.25) |
| Not living alone | -8,55 (14.17) | -3,69 (10.87) | -2,06 (8.18) | -3,06 (10.53) | -5,66 (13.46) | -10,54 (15.86) | -9,78 (16.38) | -10,17 (14.31) | -6,69 (9.40) | -0,82 (1.60) | -0,20 (1.14) |
| Living alone | -12,40 (17.75) | -4,75 (12.69) | -4,97 (11.73) | -5,98 (12.20) | -10,79 (18.18) | -10,00 (16.73) | -13,74 (20.50) | -10,95 (15.64) | -9,19 (11.30) | -1,23 (1.63) | -0,46 (1.27) |
| Not Living in a care facility | -9,50 (14.66) | -2,71 (8.83) | -2,73 (9.24) | -3,60 (10.46) | -6,67 (14.35) | -11,31 (16.45) | -11,10 (17.67) | -11,19 (14.73) | -7,35 (9.73) | -0,92 (1.57) | -0,26 (1.20) |
| Living in a care facility | -10,17 (18.06) | -9,50 (17.91) | -3,50 (10.01) | -5,08 (13.47) | -8,91 (17.89) | -6,42 (13.83) | -10,00 (17.87) | -6,92 (14.01) | -7,55 (11.24) | -1,01 (1.78) | -0,34 (1.10) |

*Note*. Income = monthly individual net income; CFQ = Cognitive Failures Questionnaire total score; GDS-15 = Geriatric Depression Scale-15 total score; LSNS-6 = Lubben Social Network Scale-6 total score; BRS = Brief Resilience Scale mean score; PWI-A = Personal Wellbeing Index – Adults; PWI-A1 to PWI-A8 = difference scores between the reports on the individual PWI-A items (respectively, general life satisfaction, standard of living, health, achieving in life, relationships, safety, community connectedness and future security) during and before the COVID-19 period; PWI-A_total = difference scores between the PWI-A total scores (i.e., subjective wellbeing) during and before the COVID-19 period; Activity and Sleep = difference scores between the reports for activity level and sleep quality during and before the COVID-19 period; *** *p<*.001; ** *p<*.01; * *p<*.05.

**Supplementary Table 3**

*Pearson correlations between the continuous predictors (age, income, CFQ, GDS-15, LSNS-6 and BRS total scores) and the subjective cognitive change questions. Means (SD) for the subjective cognitive change questions for each level of the categorical predictors (i.e., gender, whether the participant lived alone or not and whether the participant lived in a care facility or not).*

|  | Problems with | | | | | |
| --- | --- | --- | --- | --- | --- | --- |
|  | cognitive functioning | remembering | concentration | doing two things at the same time | recalling | forgetfulness |
| Age | -0.014 | -.034 | .11** | .047 | .038 | -.030 |
| Income | .054 | .066 | .087* | .050 | .078 | .067 |
| CFQ | -.29*** | -.32*** | -.29*** | -.21*** | -.32*** | -.31*** |
| GDS-15 | -.35*** | -.26*** | -.33*** | -.18*** | -.26*** | -.31*** |
| LSNS-6 | .11** | .033 | .061 | .015 | .080* | .10* |
| BRS | .17*** | .13** | .13** | .08 | .17*** | .17*** |
| Male | 1,95 (0.30) | 2,94 (0.35) | 2,94 (0.35) | 2,96 (0.33) | 2,94 (0.38) | 2,94 (0.39) |
| Female | 1,94 (0.32) | 2,92 (0.44) | 2,88 (0.50) | 2,98 (0.36) | 2,91 (0.42) | 2,92 (0.41) |
| Not living alone | 1,95 (0.30) | 2,93 (0.37) | 2,92 (0.41) | 2,97 (0.32) | 2,93 (0.37) | 2,93 (0.38) |
| Living alone | 1,92 (0.34) | 2,94 (0.49) | 2,87 (0.52) | 2,99 (0.40) | 2,89 (0.50) | 2,91 (0.46) |
| Not living in a care facility | 1,95 (0.32) | 2,93 (0.41) | 2,89 (0.46) | 2,96 (0.33) | 2,91 (0.41) | 2,93 (0.40) |
| Living in a care facility | 1,92 (0.28) | 2,93 (0.38) | 2,98 (0.35) | 3,04 (0.40) | 2,95 (0.41) | 2,89 (0.43) |

*Note*. Income = monthly individual net income; CFQ = Cognitive Failures Questionnaire total score; GDS-15 = Geriatric Depression Scale-15 total score; LSNS-6 = Lubben Social Network Scale-6 total score; BRS = Brief Resilience Scale mean score; *** *p<*.001; ** *p<*.01; * *p<*.05.

**Supplementary Figure 1**

*Matrix scatterplot containing all two-by-two scatter plots between age, CFQ, GDS-15, PWI-A_pre, PWI-A_current, LSNS-6 and BRS. Red lines indicate fitted linear regression lines.*


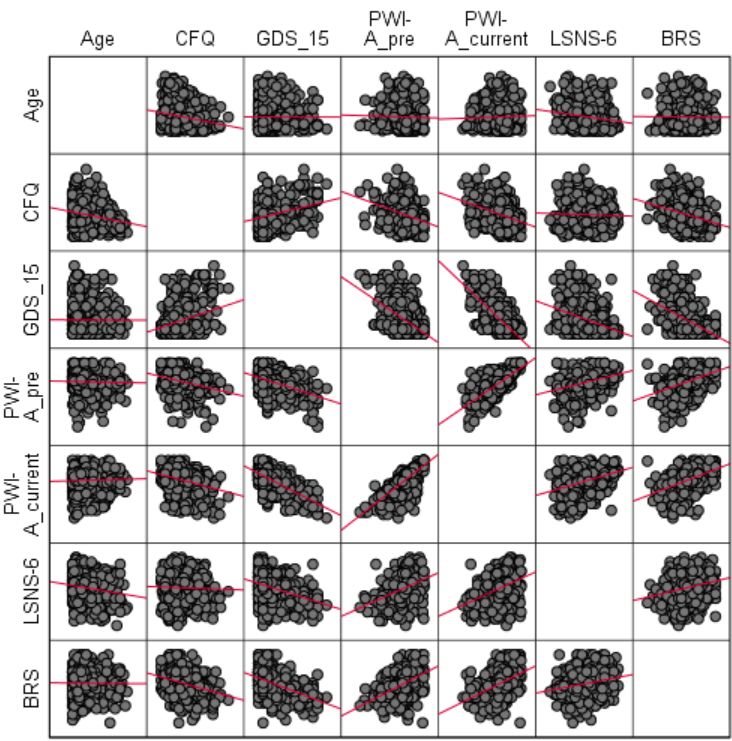

Supplement: Supplementary file 1 — Supplementary Information. [file 41598_2021_84127_MOESM1_ESM.docx]
